# Impact of action observation therapy on motor and cognitive outcomes in older adults with mild cognitive impairment: a randomized controlled study

**DOI:** 10.3389/fpubh.2025.1518092

**Published:** 2025-02-21

**Authors:** Miriam Martin-Blazquez, M. Dolores Sosa-Reina, Angye Micaela Andrade-Granda, Ismael Sanz-Esteban, Javier López-Ruiz, Cecilia Estrada Barranco

**Affiliations:** ^1^Physiotherapy Department, Faculty of Sport Sciences, Universidad Europea de Madrid, Madrid, Spain; ^2^Neurosciences and Physical Therapy Research Group, Faculty of Sport Sciences, Universidad Europea de Madrid, Madrid, Spain; ^3^Musculoskeletal Pain and Motor Control Research Group, Faculty of Sport Sciences, Universidad Europea de Madrid, Madrid, Spain

**Keywords:** action observation therapy, older adults, mild cognitive impairment, mirror neurons, physical training

## Abstract

**Background:**

Mild Cognitive Impairment (MCI) affects both cognitive and motor function, increasing the risk of immobility, falls, and compromising independence. The aim of this study is to determine whether implementing Action Observation Therapy (AOT) in a population with MCI would yield positive outcomes in cognitive status, in activities of daily living (ADLs), upper limb (UL) functionality, gait and balance, and if these results differ based on the observation modality employed.

**Methods:**

Thirty participants, aged 65 and above with MCI, were assigned to three groups: therapist observation group (TOG), peer observation group (POG), and control group (CG). The intervention comprised physical and cognitive exercises over 5 weeks, with assessments before and after.

**Results:**

significant differences in post-intervention improvement were found between the groups, excluding the Box and block test. No significant differences were found between the TOG and POG in any improvement variable. Significant differences were found between the CG and the observation groups.

**Conclusions:**

The intervention with AOT proved beneficial for individuals with MCI, yielding significant results both when observing the therapist and when observing a peer compared to the CG, in the domains: cognition, ADLs, gait and balance.

## Introduction

Mild cognitive impairment (MCI) is defined as a “syndrome characterized by cognitive deficits greater than expected for the individual's age and educational level, without significant impairment in activities of daily living (ADLs), and without meeting criteria for dementia. Memory impairment is the primary issue, but there may also be deterioration in other cognitive areas” (1).

According to the World Health Organization (WHO), in 2014 there were 5.0 million adults aged 65 and older with cognitive impairment (CI), and it is projected that by 2060, this number will almost reach 14 million (2). The progressive aging of the population suggests a rising incidence of MCI, posing significant challenges for society (3).

Limitations in executive function, present in patients with MCI, can result in a reduction in physical mobility over time due to a decrease in the ability to perform complex and ADLs (4). This is achieved through a combination of motor, cognitive, and socioemotional skills (5). MCI decrease independence in ADLs, leading to increased immobility and social isolation (6, 7). Additionally, cognitive functions have been linked to motor functions of the upper limbs (UL), including fine motor dexterity, alternate forearm movements, and bimanual coordination (8). Therefore, patients with MCI often present impairments in UL function. On the other hand, gait and balance dysfunctions are common in individuals with CI. Among the most frequent parameters are decreased walking speed, reduced stride length, and changes in swing time, among others (9). These alterations significantly increase the risk of falls. Approximately 60% of individuals with CI experience falls annually, representing double the incidence compared to individuals of the same age without CI (10, 11).

Intervention based on the combination of physical and cognitive training in a functional context can help improve dysfunction caused by MCI. Therefore, it seems appropriate to seek rehabilitation tools that integrate these requirements for the improvement or preservation of skills in individuals with MCI.

The Action Observation Therapy (AOT) involves observing a motor gesture performed by another individual and then proceeding to replicate the same movement (12). This technique has been developed as a physical rehabilitation approach that promotes brain plasticity by activating the mirror neuron system (MNS). The MNS is a network of specialized neurons that are activated both during the execution of a movement and when observing the same movement performed by others. This dual activation facilitates the imitation and learning of motor skills by linking observation with motor execution. Moreover, the MNS is thought to play a crucial role in enhancing cognitive functions, such as memory and attention, as well as emotional processes, such as empathy and social understanding (13).

Age- and sex-related variations in the activation of the MNS have been reported, suggesting that these factors may influence the effectiveness of interventions like AOT. For example, Pua et al. highlight how executive function mediates age-related declines in emotion recognition, with notable differences observed between sexes. These findings underline the importance of tailoring AOT interventions to address specific deficits related to executive function, which may vary with demographic factors. This theoretical basis reinforces the relevance of AOT in not only improving motor function but also in addressing cognitive and emotional deficits that are characteristic of populations with conditions like MCI (13).

AOT has proven to be an effective way to learn or enhance the performance of a specific motor skill, creating a memory trace in both adults and patients who have suffered a stroke (14). AOT has also been applied to patients with MCI, demonstrating its effectiveness in improving UL functionality and cognitive functions (15, 16).

While AOT has demonstrated its effectiveness in various populations, there remains uncertainty about which modality, therapist or peer observation, is most effective, particularly for older adults with MCI. Previous research, such as Naura et al. (17), has shown that in children with cerebral palsy (CP), motor learning is enhanced when observing peers with similar conditions rather than therapists or individuals without impairments. This finding underscores the potential role of social and cognitive relatability in maximizing AOT outcomes. However, these findings have not been explored in older adults with MCI, who present distinct cognitive and motor challenges compared to other populations.

This study addresses a critical gap in literature by investigating the differential impacts of therapist vs. peer observation modalities in AOT for older adults with MCI. Specifically, we aim to determine whether AOT interventions can improve cognitive status, UL function, ADLs, gait, and balance, and whether these outcomes differ depending on the observation modality.

## Methodology

### Study type

This randomized, block-structured, analytical, and longitudinal experimental study was conducted in three senior care facilities in Madrid, Spain. Approval was obtained from the ethics committee of the Hospital Clínico San Carlos (code: 23/122-E), and the study was registered in the Clinical Trials registry (NCT05934344). The study adhered to the CONSORT (18) and Helsinki guidelines to ensure ethical (19) and methodological rigor.

### Participants

Eligible participants were adults aged 65 and older, residing in care facilities, with a Montreal Cognitive Assessment (MoCA) (20) score between 20 and 26. They had to be capable of walking independently over a 10-meter distance and free from severe pain. Exclusion criteria included uncorrectable sensory impairments (without corrective aids such as glasses or hearing aids), inability to follow simple commands, recent neurological or musculoskeletal conditions, and contraindications to physical exercise. Recruitment and initial screening were conducted by the healthcare personnel at each facility, ensuring that all selected participants met these criteria, thereby minimizing potential selection bias.

Participants diagnosed with MCI who met the inclusion criteria were recruited from three senior care facilities in Madrid, Spain. Healthcare personnel at each facility conducted initial screenings to ensure compliance with inclusion and exclusion criteria, minimizing potential selection bias. Eligible participants provided informed consent and were then randomly assigned to one of three groups using OxMaR randomization software (21). This software ensured allocation concealment and an equal distribution across groups: (1) Therapist Observation Group (TOG), (2) Peer Observation Group (POG), and (3) Control Group (CG). Dropouts were carefully documented, and an intention-to-treat analysis was employed to mitigate the impact of attrition on study results.

### Intervention

Each group underwent a 5-week intervention. In the TOG, participants observed exercises demonstrated by a therapist positioned directly in front of them. In the POG, participants observed peers without cognitive impairment performing the same exercises, also positioned in front of them to allow clear visibility. The CG continued with standard residence-based therapy, which primarily aimed at maintaining basic mobility and ADLs. The intervention sessions conducted three times per week for 20–30 min, included resistance training, balance activities, and UL exercises. The design of these sessions was inspired by the Fugl-Meyer Assessment (FMA) (22) and the Vivifrail physical training programs (23), both of which are tailored to prevent deconditioning in older adults. Importantly, the exercises were adjusted to address the specific deficits of each participant as identified through their baseline assessments using the FMA and Vivifrail tools. The 5-week intervention period was chosen based on previous studies that demonstrated meaningful improvements in motor and cognitive function within similar durations (e.g., 4–6 weeks) (24, 25).

To address potential bias due to the dropout rate in the POG, we employed intention-to-treat (ITT) analysis, where all participants were included in the statistical analysis based on their originally assigned group, regardless of whether they completed the intervention. Additionally, sensitivity analyses were performed to evaluate the robustness of the results, considering only participants who completed the intervention. These approaches aimed to minimize the impact of missing data and ensure a comprehensive evaluation of the intervention's effectiveness.

### Outcome measures

To assess cognitive function, we employed the MoCA scale (20), a tool widely recognized for its effectiveness in detecting cognitive decline (26, 27). Functionality in ADLs was evaluated using the Barthel Index (BI), which has been validated in various populations, including older adults (28, 29).

UL functionality was assessed using two validated measures: the Fugl-Meyer Assessment for Upper Extremity (FMA-UE) (22) and the Box and Block Test (BBT) (30, 31). The BBT includes normative reference values for the older adult population and has been widely used to objectively measure changes in UL motor control and performance (32).

Balance and gait were assessed using three validated tools. The Berg Balance Scale (BBS) was used to detect balance impairments and fall risk, given its reliability in older populations (33). The Time Up and Go (TUG) test provided an assessment of functional mobility and dynamic balance (34). Finally, gait speed was measured with the 10-Meter Walk Test (10MWT) in two conditions: a “normal pace” to capture habitual walking speed 10MWTN, and a “fast pace” to assess maximum gait speed capacity 10MWTF (35).

### Data analysis

The sample size was calculated using GRANMO software, based on the research by Donoghue et al. (36), targeting a minimum detectable difference of 6.3 (alpha = 0.05, beta = 0.2) with a 20% dropout rate. The Shapiro-Wilk test was performed to assess the normality of the data. As the results indicated a non-normal distribution, the Kruskal-Wallis test was employed to evaluate differences between groups. *Post-hoc* pairwise comparisons were conducted using the Mann-Whitney *U*-test.

Effect sizes were calculated for all outcomes to provide additional context, particularly for non-significant findings. Cohen's d was used to quantify the magnitude of differences between groups, with thresholds for small (0.2), medium (0.5), and large (0.8) effects. This approach allows for the interpretation of clinical relevance even in cases where statistical significance was not achieved, offering insights into the potential practical implications of the findings.

A significance level of *p* < 0.05 was set, and effect size was calculated using Cohen's d, where *d* = 0.2, *d* = 0.5, and *d* = 0.8 indicate small, medium, and large effects, respectively (30). Analyses were conducted using SPSS v.29.0.

## Results

**Figure 1:**
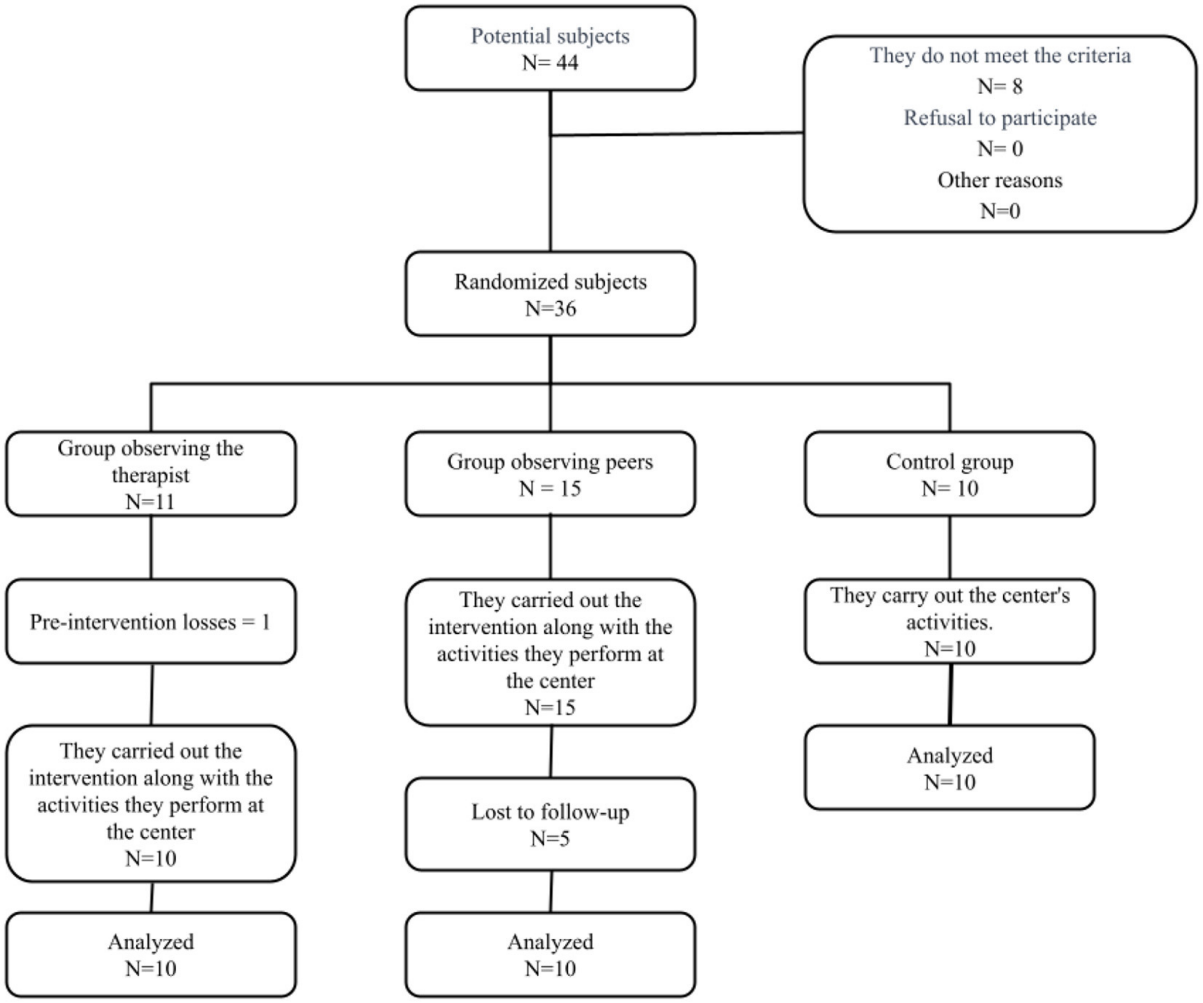
Flowchart.

A total of 36 subjects were selected from the pool of 44 eligible users who met the inclusion criteria and were grouped into 3 groups. The study concluded with 30 subjects, as there were 6 dropouts, one in the TOG before the intervention and five in the POG. In Figure 1, a flowchart is presented to illustrate the progression of participants through the study. The description of the sample is shown in Table 1.

**Table 1:** Description of the sample.

**Group**	**TOG**	**POG**	**CG**
*N*	10	10	10
Gender	1 H; 9 M	2 H; 8 M	7 H; 3 M
Age (Mean ± SD)	88.6 ± 7.18	84 (±8.03)	78.8 (±7)

TOG, Therapist Observation Group; POG, Peer Observation Group; CG, Control Group; H, Males; M, Females; SD, Standard Deviation.

The homogeneity of the groups was analyzed concerning age, gender, and initial scores on the scales used: MoCA, BI, FMA-UE, BBT, BBS, TUG, 10MWT and 10MWT fast. No significant differences were found in any of these variables between the groups before starting the intervention.

Initial and final scores were collected for all studied variables. An improvement variable for each variable was calculated by taking the difference between the final and initial scores. Significant differences were found among the three groups in the improvement variable for all studied variables, except for BBT, both right and left arm (Table 2).

**Table 2:** Results of the Kruskal-Wallis test for group differences across outcome measures.

**Measure**	**H (Kruskal-Wallis)**	**df**	***p*-value (Sig.)**
Montreal cognitive assessment (MoCA)	13.3	2	0.001
Barthel index (BI)	7.0	2	0.030
Fugl-meyer assessment (FMA)	17.2	2	< 0.001
Box and block test - left hand (BBT)	8.7	2	0.013
Box and block test - right hand (BBT)	3.7	2	0.160
Berg balance scale (BBS)	16.2	2	< 0.001
Time up and go (TUG)	15.7	2	< 0.001
10-meter walk test - normal pace (10MWTN)	16.6	2	< 0.001
10-meter walk test - fast pace (10MWTF)	8.6	2	0.013

Df, degrees of freedom.

Pairwise comparisons were conducted on the improvement variable to determine the direction of differences found between groups (Table 3). No statistically significant differences were found between the TOG and the POG in any of the improvement variables studied. Statistically significant differences were found between the CG and the TOG in the improvement variables of MoCA, FMA, BBT right, BBT left, TUG, 10MWTN, and 10MWTF. However, no statistically significant differences were found in the BI. In the comparison between the POG and the CG, statistically significant differences were found in the improvement variables of MoCA, FMA, BBS, TUG, 10MWTN, and 10MWTF. However, no statistically significant differences were found in the BI and BBT right and left arm.

**Table 3:** Pairwise comparisons of improvement variables between groups.

**Measure**	**TOG M (IQR)**	**POG M (IQR)**	**CG M (IQR)**	**TOG vs. POG**	**TOG vs. CG**	**POG vs. CG**	**TOG vs. CG Cohen's d**	**POG vs. CG Cohen's d**
MoCA	2.5 (1.0, 4.0)	2.5 (1.25, 3.75)	0.0 (−0.5, 0.5)	49 (=0.97)	6 (< 0.001)	13 (=0.004)	1.51	1.77
BI	2.5 (−0.62, 6.87)	2.5 (0.0, 5.0)	0.0 (0.0, 0.0)	47.5 (=0.83)	25 (=0.13)	25 (=0.063)	NA	NA
FMA	23.5 (17.62, 29.37)	29.0 (9.12, 48.87)	1.0 (−1.12, 5.37)	41 (=0.53)	0 (< 0.001)	6 (< 0.001)	3.44	1.34
Left BBT	4.0 (2.75, 5.25)	4.5 (−2.25, 19.25)	0.0 (−1.25, 1.25)	49.5 (=0.97)	10 (=0.002)	24 (=0.05)	2.16	0.58
Right BBT	6.0 (2.12, 9.87)	2.0 (−4.75, 6.75)	0.5 (−0.12, 0.62)	44.5 (=0.68)	23 (=0.04)	36 (=0.315)	1.34	NA
BBS	8.5 (6.0, 11.0)	9.0 (4.87, 13.12)	0.0 (−1.62, 1.62)	49 (=0.97)	0 (< 0.001)	9 (=0.001)	2.72	1.94
TUG	−5.8 (−8.4, −3.2)	−5.1 (−7.63, −2.57)	−0.34 (−1.13, 1.25)	45 (=0.7)	5 (< 0.001)	5 (< 0.001)	−1.92	−1.71
10MWTN	−2.2 (−3.74, 0.88)	−3.08 (−5.18, −0.98)	−0.4 (−0.95, 0.15)	29 (=0.11)	7 (=0.001)	4 (< 0.001)	−1.05	−1.18
10MWTF	−1.15 (−2.0, 0.5)	−1.7 (−2.58, −0.82)	−0.145 (−0.98, 1.52)	38 (=0.36)	19 (=0.019)	16 (=0.009)	−0.82	−1.22

Results are expressed in Mann-Whitney U (*p*-value); M: Median; IQR: interquartile range; TOG: Therapist Observation Group, POG: Peer Observation Group, CG: Control Group, MoCA: Montreal Cognitive Assessment, BI: Barthel Index, FMA: Fugl Meyer Assessment, BBT: Box and block test, BBS: Berg Balance Scale, TUG: Time Up and Go, 10MWTN: 10-Meter Walk Test, 10MWTF: Ten-Meter Walk Test (Fast). NA: not available. Statistical Significance Level: *p* = 0.05.

## Discussion

The objectives of this study were to assess whether there are changes in cognitive status, ADLs, UL functionality, balance and gait in patients with MCI after an intervention with AOT. Additionally, the study aimed to determine if there are differences between observing a therapist or a peer without MCI. Statistically significant improvements were found in four domains: cognition, ADLs, gait and balance, in both the TOG and the POG, in contrast to the CG. Overall, the results indicate that AOT, implemented three times per week over a 5-week period, was sufficient to elicit significant improvements in both intervention groups. Furthermore, the inclusion of a CG provided critical insights, as no significant changes were observed following conventional therapy in this group, reinforcing the added value of AOT interventions.

Our results are consistent with evidence supporting the effectiveness of AOT in promoting both motor and cognitive recovery across diverse populations. As highlighted in a recent systematic review, AOT shows promise as an effective strategy in rehabilitation programs for stroke patients, enhancing the functionality of the paralyzed UL and facilitating the recovery of maximum independence in ADLs (25, 37). Additionally, it appears to be a promising approach for individuals with MCI (15). Furthermore, engaging the MNS through observation and imitation of actions, the use of AOT in children diagnosed with CP has demonstrated its ability to enhance motor function, increase the spontaneous use of the affected UL, and promote functional participation in ADLs (38–40). These findings suggest that the mechanisms underlying AOT, particularly MNS activation, play a critical role in driving neuroplastic changes and facilitating motor learning across diverse populations.

Moreover, the role of the MNS in other movement disorders, such as Parkinson's disease, presents a compelling avenue for future research. Individuals with Parkinson's disease often exhibit hypomimia, or reduced facial expressivity, and impairments in emotion recognition, particularly disgust, which may be linked to MNS dysfunction (41, 42). These deficits could potentially influence the outcomes of AOT interventions, emphasizing the need to tailor such therapies to the specific neurocognitive profiles of older adults with movement disorders.

The intervention duration chosen for this study reflects a careful balance between achieving measurable outcomes and addressing practical considerations such as participant adherence and program feasibility in residential or community settings (10, 24). Consistent with findings from other studies (14, 23, 25), our research applied a tailored adjustment of exercise frequency, duration, and intensity to optimize outcomes. Sessions lasting 20–30 min, conducted three times per week over a 5-week period, appear to represent an optimal dosage for achieving significant improvements in individuals with MCI, as observed in stroke patients (14, 25). Identifying the most suitable exercise protocols for individuals with MCI remains essential for ensuring specific and effective results. Additionally, community-based approaches that integrate AOT within group exercises have shown to further enhance functional outcomes, highlighting the need to optimize intervention duration and delivery to maximize benefits for individuals with MCI, who often have limited physical and cognitive reserves (11, 29, 43).

Regarding MoCA improvement in the three groups, they support the notion that gait and balance capacity may be crucial for potential improvements in cognitive skills. This is likely that exercise evokes significant benefits in functional changes in the brain, reductions in white matter lesions, and decreases in atrophy across all age groups (24).

Results in this study showed improvements in both the TOG and POG compared to the CG, although these differences did not reach statistical significance (*p* = 0.13 and *p* = 0.063, respectively). The median BI improvement in the intervention groups (TOG: 2.5; POG: 2.5) highlights a trend toward functional gains in ADLs, while no changes were observed in the CG (median: 0.0). However, the BI's limited sensitivity to detect subtle functional improvements, particularly in populations with MCI, may have contributed to the lack of significant findings. Despite its widespread use, adequate reliability and validity (28, 44, 45), our results align with critiques of the BI as a general measure that may not fully capture nuanced changes in functional capacity in population with MCI (46, 47).

The most significant improvements were observed in the FMA in both experimental groups, which can be attributed to receiving specific therapy for the UL with a progression of several sessions per week. In the study by Fu et al., where AOT intervention was also conducted, significant changes in FMA were observed (48). In the study by Mao et al., after 8 consecutive weeks of training, both groups showed significant improvements in UL motor function. The mirror neuron system group, which underwent AOT therapy, exhibited significantly improved motor and cognitive function of the UL compared to the CG (49). Furthermore, we also observed significant differences and improvements in BBT scores when comparing the AOT groups (TOG and POG) with the CG. These findings align with previous studies that highlight the efficacy of AOT in enhancing UL functionality in stroke patients (31, 50).

Research support that gait and balance tend to deteriorate in individuals with MCI (51). The present study demonstrates that physical activity can help improve gait and balance in individuals with MCI. Health professionals could utilize AOT as an accessible and cost-effective therapeutic strategy to address gait and balance issues in individuals with MCI. The exercises employed in this study (Appendix 1) were well-suited to the participants, leading to significant improvements in the TUG, which evaluates the time required to walk a specific distance and make turns. Although the intervention did not include specific exercises to enhance walking speed due to safety concerns and participants' abilities, notable improvements were observed in the 10MWT, with reduced times to cover the set distance. These changes likely reflect enhanced balance, functionality, and fatigue resistance (34).

In our hypothesis, we suggested that therapy would be more effective when learning occurred among peers, as the MNS appears to be activated by mechanisms related to empathy, with increased activity when the observer can identify shared characteristics with the individual being observed (49, 52, 53). According to our results, although both groups show improvement, it is greater in the group that observes the therapist. These results differ from those obtained by Nuara et al. (17), where children with CP learned more by imitating other children than an expert therapist. Conversely, our results align with those of Rohbanfard et al. (54), who concluded that observing an expert is more effective than observing a novice.

The lack of superiority observed in the POG compared to the TOG may be attributed to individual differences such as fatigue, age-related cognitive and physical changes. Older adults with MCI are particularly prone to cognitive fatigue, which can limit their ability to engage effectively with the additional cognitive demands posed by peer observation (55). Furthermore, discrepancies in movement execution or speed among peers may increase the cognitive load, potentially diminishing the benefits of imitation-based learning (56). These findings emphasize the need structured guidance, clear demonstrations, and perceived authority in therapeutic interactions might also play a critical role in this population, where cognitive and physical reserves are limited (57, 58). Further research is needed to explore these dynamics in depth and to assess whether modifications to peer observation protocols could mitigate these factors.

Pairing in the POG was done randomly. Group work has benefits, as participants encourage and motivate each other to perform exercises (59, 60). The significant improvements observed in both intervention groups compared to the CG highlight the value of group-based approaches. While therapist-guided AOT can provide clear benefits, it requires trained professionals, making it resource-intensive. Peer observation protocols offer a cost-effective alternative when combined with proper training and supervision to ensure safety and engagement. Utilizing peer facilitators in community or residential settings could expand access to rehabilitation while reducing the need for individual therapist time. Further research should focus on optimizing these models and evaluating their long-term effects on functional and cognitive outcomes in individuals with MCI (49, 59, 60).

By broadening the comparison to these populations, our findings underscore the versatility of AOT as a rehabilitation strategy. While our study focuses on older adults with MCI, the underlying principles of MNS activation and its capacity to enhance motor and cognitive functions appear to be universally applicable, if interventions are adapted to the unique characteristics and challenges of each population.

The limitations of this study include the small sample size, which may restrict the generalizability of the findings. Larger-scale studies are necessary to confirm these outcomes and provide more robust evidence. Additionally, the absence of long-term follow-up limits our understanding of the sustainability of the observed improvements in gait and balance. Another limitation lies in the use of the Barthel Index, which may lack sensitivity to detect subtle changes in functional capabilities, particularly in older adults. To address this, future research should consider integrating complementary measures, such as social functioning scales for specific populations like Parkinson's disease (61), or frameworks such as the International Classification of Functioning, Disability, and Health (ICF) for a more comprehensive assessment of disability in older adults (47). Finally, incorporating integrative measurement approaches, as suggested in transitions from hospital to home care for older adults (46), may further enhance the sensitivity and applicability of functional assessments in this population.

## Conclusions

Our results indicate that AOT is an effective intervention for improving motor and cognitive function in older adults with MCI. The two observation modalities studied: The use of both therapist and peer observation modalities produced significant improvements in functional outcomes, suggesting the flexibility and adaptability of AOT as a therapeutic approach. These results support the inclusion of AOT in rehabilitation programs for individuals with MCI, potentially addressing key areas such as cognitive impairment and fall risk. Future studies with larger cohorts and longer follow-up periods are warranted to confirm these findings and establish the long-term benefits of AOT in this population.

## Data Availability

The raw data supporting the conclusions of this article will be made available by the authors, without undue reservation.
