# Supplementary material for: Impact of action observation therapy on motor and cognitive outcomes in older adults with mild cognitive impairment: a randomized controlled study
Source: Front Public Health. 2025 Feb 21;13:1518092. doi: 10.3389/fpubh.2025.1518092 (PMC11885144; doi:10.3389/fpubh.2025.1518092)
Supplement: Supplementary file 1 [file Data_Sheet_1.pdf]

Appendix 1. Table of exercises per week

**Week 1**	Global exercises, upper limb mobilizations, active mobilization of lower limbs, and basic physical preparation exercises, seated balance exercises, and gentle walking.
**Week 2**	Exercises involving midline crossing + synergies + global mobilizations in upper limbs, active mobilization of lower limbs and trunk, seated balance exercises, functional exercises like touching their toes, and gentle walking.
**Week 3**	Strength + endurance exercises for upper limbs, resisted exercises for lower limbs with a yellow elastic band, trunk exercises, functional exercises like making a full turn, balance exercises in standing position, and walking.
**Week 4**	Fine motor skills exercises (grasps and pinches) with coordination and speed in upper limbs, resisted exercises with a yellow elastic band maintaining the position for 5 seconds, functional exercises, stability and balance exercises in standing position, walking.
**Week 5**	Combination of exercises from weeks 1 to 4.
