# Supplementary material for: Impact of action observation therapy on motor and cognitive outcomes in older adults with mild cognitive impairment: a randomized controlled study
Source: Front Public Health. 2025 Feb 21;13:1518092. doi: 10.3389/fpubh.2025.1518092 (PMC11885144; doi:10.3389/fpubh.2025.1518092)
Supplement: Supplementary file 2 [file Data_Sheet_2.pdf]

Day	Monday	Wednesday	Friday
**Week 1**	**Exercises with Verbal Cues (Patients Seated)**	**12 repetitions of each exercise**	**16 repetitions of each exercise**
**Goal:** Mobilization and coordination.	- Raise arms as high as possible.<br>- Move both arms behind the chair backrest.<br>- Elevate arms (right and left) from the sides as high as possible.<br>- Make the largest circles possible with stretched arms.<br>- With elbows at the sides of the body, rotate the hand outward (external rotation), and bring the arm to touch the torso without lifting the arm. Alternate arms.<br>- Perform elbow flexion and extension.<br>- Rotate wrists palms upward and downward with elbows close to the body.<br>- Perform wrist flexion and extension with elbows close to the body.	- Raise arms as high as possible with a ball between hands.<br>- Move both arms behind the chair backrest.<br>- Elevate arms from the sides (right and left) with a ball to transfer it from one hand to the other.<br>- Perform large arm circles with arms extended.<br>- External rotation with a ball and transfer it to the other hand.<br>- Perform elbow flexion and extension while holding a ball.<br>- Wrist movements with the ball (palms up and down).<br>- Wrist flexion and extension with a ball.<br>- Perform ulnar and radial deviations using the ball.<br>- Open and close fingers and hands.	- Raise arms with a stick between hands.<br>- Move both arms behind the chair backrest.<br>- Elevate arms (right and left) with a stick or band to transfer it.<br>- Large arm circles with extended arms.<br>- Perform rotations while holding the stick.<br>- Wrist movements with the stick.<br>- Ulnar and radial deviations.<br>- Open and close fingers and hands.

	- Perform ulnar and radial deviations (as if waving or caressing something).<br>- Make 8 wrist circles to the right and then to the left.<br>- Open and close fingers and hands.		
**Week 2**	**Exercises with Midline Crossing + Synergies**	**Midline Crossing Coordination**	**Midline Crossing and Strength**
**Goal:** Enhance coordination and range.	- Shoulder flexion/extension and abduction.<br>- Alternate maximum flexion and relaxation of arms.<br>- Bring palms together at 90º abduction.<br>- Cross arms at the midline.<br>- Diagonal upper/lower arm movements.<br>- Pick objects diagonally (right to left).<br>- Boxing motions with arms.<br>- Midline object transfer on a table.	- Mobilization of fingers, wrists, and elbows.<br>- Diagonal picking tasks.<br>- Boxing motions.<br>- Midline object placement and alternation (colored balls).<br>- Playful tasks involving crossing arms over the head and to the opposite side.<br>- Rapid hand switching tasks.	- Shoulder mobilization: flexion/extension, abduction, circumduction.<br>- Crossing objects diagonally.<br>- Boxing motions.<br>- Reorganizing midline placement tasks (color-coded blocks).<br>- Coordination and fine motor control games.<br>- Flexion/extension exercises with objects.
**Week 3**	**Strength and Resistance Training**	**Resistance Band Training**	**Progressive Resistance Training**

**Goal:** Increase muscle endurance.	- Shoulder flexion to maximum range (weights or bands).<br>- Abduction with weights.<br>- Internal/external rotation.<br>- Wrist flexion/extension.<br>- Ulnar and radial deviations.<br>- Finger extension resistance.<br>- Ball squeezes for grip strengthening.	- Shoulder flexion to maximum range.<br>- Abduction with weights.<br>- Internal/external rotation.<br>- Wrist flexion/extension.<br>- Finger grip with increasing resistance.<br>- Coordination with balls.	- Shoulder flexion to lateral elevation.<br>- Abduction with resistance.<br>- Rotational strength tasks.<br>- Wrist movements and fine motor endurance.<br>- Progressive strength building.
**Week 4**	**Fine Motor Skills and Coordination**	**Daily Life Skill Tasks**	**Object Manipulation and Coordination Games**
**Goal:** Develop fine motor skills.	- Drawing exercises.<br>- Cutting simple shapes.<br>- Picking up small objects (beans, coins).<br>- Stacking and unstacking cubes.<br>- Stringing beads onto a cord.	- Buttoning and unbuttoning shirts.<br>- Zipping and unzipping tasks.<br>- Opening/closing various lids.<br>- Modeling clay work.<br>- Placing pegs on a board.	- Clothespin placement and usage.<br>- Quick object placement games (beans into bottles).<br>- Repositioning tasks for coordination.<br>- Page-turning practice.
**Week 5**	**Mixed Exercises**	**Combined Exercises**	**Integrated Coordination Training**
**Goal:** Combine mobility, strength, and	- Shoulder and elbow range of motion.<br>- Wrist and finger	- Abduction/adduction tasks with weights.<br>- Midline crossing	- Progressive tasks combining strength, coordination, and

fine motor control.	strengthening.<br>- Midline coordination games.<br>- Fine motor skill exercises with buttons, zippers, and clothespins.	games.<br>- Fine motor tasks using paper textures.<br>- Strength and endurance combinations.	speed.<br>- Practical tasks like lifting, moving objects, and page-turning.<br>- Fine motor coordination with everyday objects.
